# How Health Impact Assessments (HIAs) Help Us to Select the Public Health Policies Most Likely to Maximise Health Gain, on the Basis of Best Public Health Science

**DOI:** 10.3934/publichealth.2016.2.235

**Published:** 2016-04-23

**Authors:** Hilary A Dreaves

**Affiliations:** Department of Public Health and Policy, University of Liverpool, UK

**Keywords:** HIA, air pollution, climate change, sustainability, environmental assessment

## Abstract

Health Impact Assessment (HIA) is a decision support tool intended to present timely, evidence–based recommendations to decision makers in all sections of society in order to accentuate potential positive health and well-being impacts (and mitigate potential negative impacts) of policies, plans (including local and neighbourhood plans), programmes and projects (including infrastructure and local development proposals), in order to reduce health inequalities/disparities. HIA is a well established and proven means of linking research evidence from public health and the environmental sciences with equitable decision making processes at all levels, from local to global. It may also provide a platform for examination of research proposals to strengthen the impact statement therein, identifying potential for future public benefit. This paper highlights some of the main drivers for a timely re-emphasis on the use of best scientific evidence and systematic HIA to inform decision making for future public benefit, citing the example of air pollution.

## 1. Background

**Figure 1.**
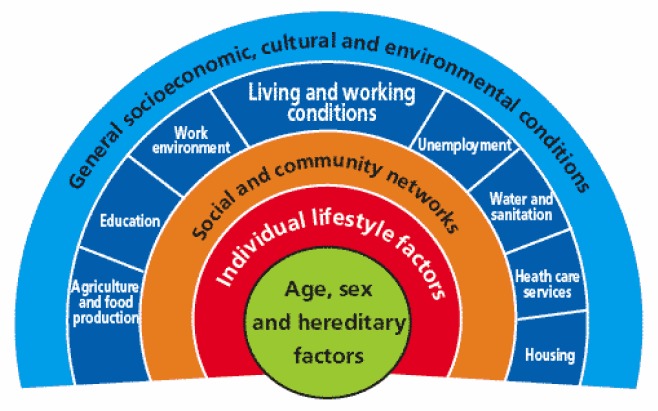
A socio-environmental model of health Source: Dahlgren and Whitehead (1991). Policies and strategies to promote social equity in health. Stockholm: Institute of Future Studies

Health Impact Assessment (HIA) was first defined in 1999 as “A combination of procedures, methods and tools by which a policy, programme or project may be judged as to its potential effects on the health of a population and the distribution of those effects within the population” [1]. The methodology is underpinned by the ubiquitous socioeconomic model of health shown in Figure 1, also commonly known as the “rainbow model” illustrating the wider determinants of health [2].

## 2. The roots of HIA, in healthy public policy and environmental assessment

The two main roots of HIA methodology were and continue to be firstly, response to concerns about hazardous environmental events affecting population health (examined through the lens of Environmental Assessment methods) and secondly, public health and health promotion disciplines that seek to develop healthy public policies globally, to protect and improve population health and well-being and reduce health inequalities or disparities (through the lens of HIA). HIA and all its' various iterations, such as those focussing on equity and mental well-being, continues to be advocated globally as a tool for the effective implementation of Health in All Policies [3], a World Health Organisation (WHO) initiative intended to support the development of healthy public policies, providing a tangible way for partners (at all levels and across organisations and sectors) to actually work together rather than just talking about working together [4].

The emergence of these two separate disciplines in parallel, each in their various formats and iterations, each founded upon (different) sound and robust scientific evidence bases has resulted in overlaps in methodology, but in practice and implementation, few links between them [5]. Nor has the emergence of separate nomenclatures and terminologies assisted in encouraging a closer and now ever more pressing need globally and nationally for shared understanding and joint, efficient, working in seeking to address prescient matters such as global warming, climate change, energy and infrastructure sustainability, preparedness for emergency and disastrous events, in combination with the demands of an increasing and ageing population and against a background of austerity measures in all sectors.

## 3. Consideration of Individual and Population; Risk and Benefit

For example, discussing at some length the connotations of language used in the two disciplines, particularly the word risk (bringing the notion of hazard related usually to a *substance* and thus inferring the possibility of quantifying the probability of exposure and severity of consequences), it has been noted that while risk assessments can indeed be descriptive and qualitative, this is not always helpful to lovers of quantitative data (and consequently those who seek to monetise the estimates made in their analysis)[6]. Impact assessments are aimed at structuring and supporting the development of “better” (ie of greater public benefit, particularly for those with least equity in society) policies, plans, projects and programmes relative to the *wider determinants* of health (Figure 1). The credibility of risk assessment can therefore be challenged as being unnecessarily complex and not well connected in reality to the needs and demands of decision making processes. Additionally, the lack of stakeholder involvement and participation in risk assessment (a characteristic of most HIAs) is a pitfall that reduces reliability and transparency of outputs; it needs to relate to the needs of decision makers earlier and be more holistic than cause and effect; the level of detail needs to be adjusted to decision making needs and in an era of civic engagement, co-design, co-production, community resilience, etc, it needs stakeholder participation (rather than merely statutory consultation or engagement processes) at all stages.

## 4. Strengthening methodology

**Figure 2.**
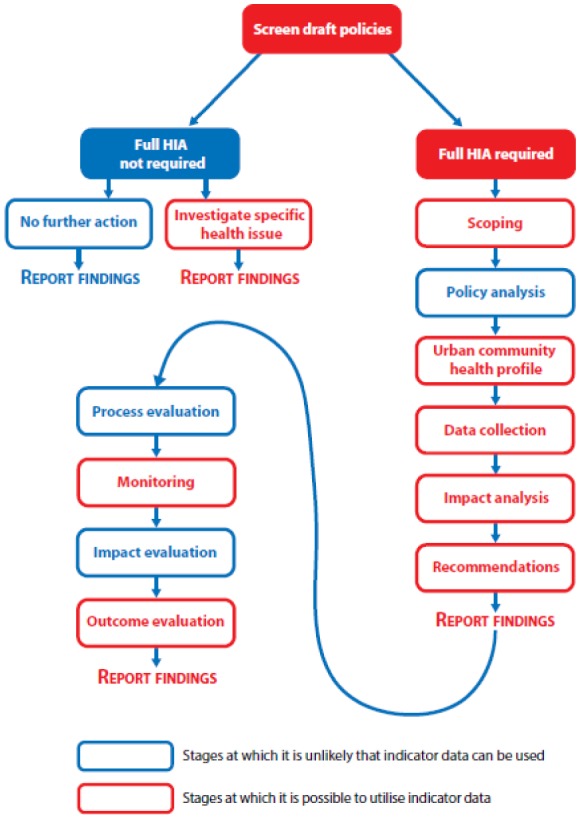
UrHIA methodological framework Source: Dreaves H, Pennington A, Scott-Samuel A (2015) Urban Health Impact Assessment methodology (UrHIA). Liverpool: IMPACT, University of Liverpool. http://www.liv.ac.uk/media/livacuk/instituteofpsychology/Urban_HIA_guide_2015.pdf

There are many methodological iterations of HIA available in the literature, covering a wide continuum of practice [7], previously described as ranging from “tight” risk reduction approaches based upon a bio-medical model of health to “broad” approaches, based upon the wider determinants of health model[8]. A recent iteration of HIA methodology (Figure 2) was developed to better link routinely available summary population statistics to policy and decision makers in a timely and meaningful way through the shared participative HIA experience [7].

## 5. Using public health science in impact assessments, for example, air pollution

The pressing need to bring a greater shared understanding of the evidence bases available to the now very separate impact assessment disciplines is perhaps best illustrated by consideration of the potential health impacts of air pollution, both ambient and household, relative to regulatory requirements. In the environmental assessment paradigm, projects and proposed developments are assessed in very considerable detail with regard to only the potential negative effects of (along with other matters) the statutory nuisances of air, noise and water, within the statutory requirements of an EU Directive on Environmental Impact Assessment (EIA). From the assessment, mitigation measures are proposed for achievement of compliance, consent or approval, i.e. the avoidance of legal infringement and consequent financial penalties, with management plans devised for their implementation. Statutory legislation requires air pollution monitoring of particulate matter (PM) of different sizes, i.e. slightly larger particles (PM 10) and slightly smaller particles (PM 2.5) found in the air [7]. Routine monitoring at PM 2.5 is yet to be wholly implemented in all EU countries. In UK local authorities, further complexity comes from a focus on nitrous oxides monitoring for environmental protection requirements by their environmental health departments, while their public health teams focus on PM 2.5 as required in the latest public health outcomes framework. Provided the project or proposed development meets these requirements, all is regarded as “well” and planning approval or consent processes may proceed. “Tight” biomedical HIAs would closely ally with the risk reduction approach and not seek to consider the wider determinants of health, the differential distribution of potential effects, any potential positive impacts, the reduction of health inequalities, strengthening of stakeholder equity in decision making, or any other potential effects, beyond those pertaining to legal requirements.

## 6. A pollution paradox?

Further updating of the EU EIA Directive (now 2014/52/EU), accepted this year and for full implementation by 2017, often adhered to internationally as good practice, requires strengthening of the consideration of health in EIA[10], moving the wider determinants HIA paradigm back into close proximity with commercial EIA practice, bringing to bear the weight of “health” evidence on the known increase in risk and susceptibility of vulnerable population subgroups, such as women and young children, to the potential effects of smaller PMs, down to the smallest nanoparticles. In future and in light of the recent World Health Assembly May 2015 resolution on air pollution [11]–[13], will it be sufficient in future to pass over the known positive health impacts of reductions in all forms of air pollution for these groups and the known interventions to reduce exposure, if a project or development proposal simply “comes in under the threshold” of air pollution monitoring as it currently stands? Shall environmental assessments (EA), formerly regarded as “passing muster” be regarded as adequate in future if they do not take account of the best public health science? What are the implications, not just for HIA, but impact assessment practice and planning consent processes globally at both national infrastructure and local levels and ultimately, of course, for the health and wellbeing of future generations?

## 7. Governance, quality and capacity building.

It has been clear since the inception of the methodology that robust, quality assured HIA, with stakeholder participation, demands a broad range of “people” skills and competencies, alongside skills such as epidemiology and statistics. Resources have never been sufficient in either health or non-health contexts, to promote carrying out detailed HIAs on every policy, project, programme, or plan—this was never the intended use of HIA. Capacity building [14] and transfer of expertise in HIA is promoted across all sectors as a means of broadening understanding of the wider determinants of health and health inequalities /disparities across the globe. There is recent evidence from Europe [15] that capacity building and training to further consideration of health in all impact assessments continue to be identified as the most important facilitators, while conversely, lack of resources and capacity building and training are barriers to it. Even in those countries with legislation for HIA, there remains a “capacity and capability premium”, creating perhaps a void in good governance and quality assurance and opportunities for the furtherment of less than robust HIA practice, something that might bring challenges to the methodology and its' practice.

There are, of course, several ways of addressing such a “void”. Academic institutions should seek to offer appropriate modules at both under and postgraduate levels in accredited cross-curricular programmes, where credits gained may be transferable for future professional portfolio development. Such modules and courses ought also to be accessible to practicing professionals as non-credit bearing continuing education and part of their professional development (and performance review, perhaps). This will however take some time to work through into professional and commercial practice which as ever, continues to be at the mercy of organisational churn, political will and resource constraints. By definition, HIA is intended to be (ideally) upstream, ex ante and prospective, a decision support tool to influence decision makers and making. HIA is necessarily imperfect; it does not validate a scientific hypothesis; it certainly is not concerned with producing reports that have no function in reality, or the production of which becomes an end in itself; that HIA cannot be tested for its' accuracy in the scientific sense; nor can it “protect” commissioners from the challenge that any proposal has damaged health; that HIA is based upon an interpretive approach to science and that it is not about stopping proposals (however inappropriate)[16].

## 8. Routine HIA screening and HIA culture

In the shorter term, then and bearing in mind that HIA is not an “expert-driven” methodology, but something that stakeholders are well able to utilise themselves, such as in community led HIAs, greater emphasis should be placed on routine, systematic HIA activity, embedding HIA culture into organisations ethos and practice. Where time and resources are of the essence, both to “do” HIA and to critically appraise reports received, routine HIA screening using validated tools or instruments specifically designed for the purpose, lends itself to evidencing (especially when placed in the public domain) that decision makers and stakeholders have indeed considered at that point in time any potential impacts on health and well-being, inequalities and equity of vulnerable sub-groups in decision making.

Where there are easily accessible, robust summaries of “what works” and for whom, i.e. the public health science evidence base on interventions and programmes, health determinants and health outcomes well studied and understood (and indeed those emerging, or re-emerging, as societies and our environment inexorably change) it would indeed be inappropriate, inefficient and wasteful to comprehensively interrogate the literature, particularly primary literature, for the purposes of HIA and informing decision makers, every single time, merely to “tick a bureaucratic box”. Better perhaps to systematically consider, the recommendations of earlier HIAs, the summary evidence gathered for them and share with colleagues and stakeholders a mutual understanding of how best the evidence can be translated into recommendations for the short, medium and longer term benefit of the population under consideration, as and when resources allow.

## 9. HIA, pure science research and public health

In conclusion, a wider use of HIA methodology, to provide a “neutral” platform for linking research findings and best evidence to policy and decision makers and making in operational settings could be a means of for example, screening research bids to strengthen their impact statement (by seeking to suggest how the anticipated findings from pure research could contribute to improvement of health and well-being and reduce inequalities, etc). In a changing Higher Education environment, this may also encourage researchers to better identify potential intellectual property (IP) for future business development.

In practice, a political perception of “success”, or lack of it, by public health professionals to achieve “closing the gap in a generation” (the strap line of the eponymous Marmot Review, i.e. WHO Commission on the Social Determinants of Health [17]) presents another timely opportunity [18] for HIA to be used to present to a new generation of policy and decision makers, known evidence from research and lessons learned from practice. Thus, systematic use of HIA presents an opportunity for them to make best use of public health science in support of evidence-based decision making, to avoid making some of the same mistakes as their predecessors.
